# Biosensor-guided improvements in salicylate production by recombinant *Escherichia coli*

**DOI:** 10.1186/s12934-019-1069-1

**Published:** 2019-01-29

**Authors:** Shuai Qian, Ye Li, Patrick C. Cirino

**Affiliations:** Department of Chemical & Biomolecular Engineering, University of Houston, S222 Engineering Building 1, Houston, TX 77204-4004 USA

**Keywords:** Metabolic engineering, Synthetic biology, Salicylate, AraC, Biosensor, High-throughput screening, Ribosome binding site

## Abstract

**Background:**

Salicylate can be biosynthesized from the common metabolic intermediate shikimate and has found applications in pharmaceuticals and in the bioplastics industry. While much metabolic engineering work focused on the shikimate pathway has led to the biosynthesis of a variety of aromatic compounds, little is known about how the relative expression levels of pathway components influence salicylate biosynthesis. Furthermore, some host strain gene deletions that improve salicylate production may be impossible to predict. Here, a salicylate-responsive transcription factor was used to optimize the expression levels of shikimate/salicylate pathway genes in recombinant *E. coli*, and to screen a chromosomal transposon insertion library for improved salicylate production.

**Results:**

A high-throughput colony screen was first developed based on a previously designed salicylate-responsive variant of the *E. coli* AraC regulatory protein (“AraC-SA”). Next, a combinatorial library was constructed comprising a series of ribosome binding site sequences corresponding to a range of predicted protein translation initiation rates, for each of six pathway genes (> 38,000 strain candidates). Screening for improved salicylate production allowed for the rapid identification of optimal gene expression patterns, conferring up to 123% improved production of salicylate in shake-flask culture. Finally, transposon mutagenesis and screening revealed that deletion of *rnd* (encoding RNase D) from the host chromosome further improved salicylate production by 27%.

**Conclusions:**

These results demonstrate the effectiveness of the salicylate sensor-based screening platform to rapidly identify beneficial gene expression patterns and gene knockout targets for improving production. Such customized high-throughput tools complement other cell factory engineering strategies. This approach can be generalized for the production of other shikimate-derived compounds.

**Electronic supplementary material:**

The online version of this article (10.1186/s12934-019-1069-1) contains supplementary material, which is available to authorized users.

## Background

Metabolic engineering facilitates improved or novel biological conversion of low-value, renewable feedstocks into higher value chemicals. By interfacing native cell metabolism with heterologous pathways, engineered microorganisms have produced a large portfolio of compounds, ranging from bulk chemicals (e.g. ethanol and butanol), to fine chemicals (e.g. aromatics) and pharmaceutical precursors (e.g. isoprenoids and alkaloids). Advances in synthetic biology are increasingly allowing for the identification and maintenance of balanced expression levels of multiple genes within a pathway [1–3]. Methods of fine-tuning and even dynamically regulating gene expression at the transcriptional and/or translational levels have been developed to improve flux through engineered pathways [4–8]. Meanwhile, metabolite-responsive transcription factors enable detection of metabolic state, where the effector molecule triggers expression of a reporter gene for in situ phenotypic screening [1].

Phenolic compounds are popular metabolic engineering targets, due to their applications as commodity chemicals [9] and pharmaceutical precursors (e.g. anti-depressant drugs, and antitumor drugs) [10]. In microbes, the shikimate pathway is the primary biosynthetic route for synthesizing aromatic amino acids and their derivatives. The key step is a condensation of glycolytic intermediate phosphoenolpyruvate (PEP) and pentose phosphate pathway intermediate erythrose-4-phosphate (E4P), generating the precursor chorismate. The shikimate pathway has been engineered to synthesize a range of natural products including flavonoids [11] and phenylpropanoids [12]. In this study, we engineered the *E. coli* shikimate pathway for production of the chorismate derivative salicylate. Salicylate can serve as a precursor to a range of pharmaceuticals such as aspirin and lamivudine (an anti-HIV drug), and is a starting material to cis–cis-muconic acid and adipic acid, the building block for nylon-6,6 and polyurethane [13]. Previous metabolic engineering work led to the functional expression of a salicylate pathway in *E. coli* [14]. Heterologous expression of isochorismate synthase (ICS encoded by *entC* from *E. coli*) and isochorismate pyruvate lyase (IPL encoded by *pchB* from *P. fluorescens* Migula) in a phenylalanine-overproducing strain resulted in the accumulation of 8.5 mM salicylate [14]. More recently, the PEP-consuming phosphotransferase system encoded by *ptsH* and *ptsI* was replaced with the heterologous expression of galactose permease and glucokinase, and genes encoding PEP-consuming pyruvate kinases (*pykA* and *pykF*) were deleted, resulting in an engineered strain that produced 80 mM salicylate in a bench-top bioreactor under optimized conditions [15].

Here we describe work to further improve salicylate production in *E. coli* by optimizing gene expression of the key enzymes in the shikimate pathway, with the help of a high-throughput sensor-reporter system. We previously engineered the *E. coli* transcription factor AraC to respond to and hence report on the presence of a number of non-native effector molecules, including triacetic acid lactone (TAL), mevalonate, and phenolic compounds [16–20]. In this study, a salicylate sensor-reporter system based on variant “AraC-SA” was rewired to control the chromosomal expression of β-galactosidase (LacZ), which cleaves X-gal and generates blue color.

**Fig. 1:**
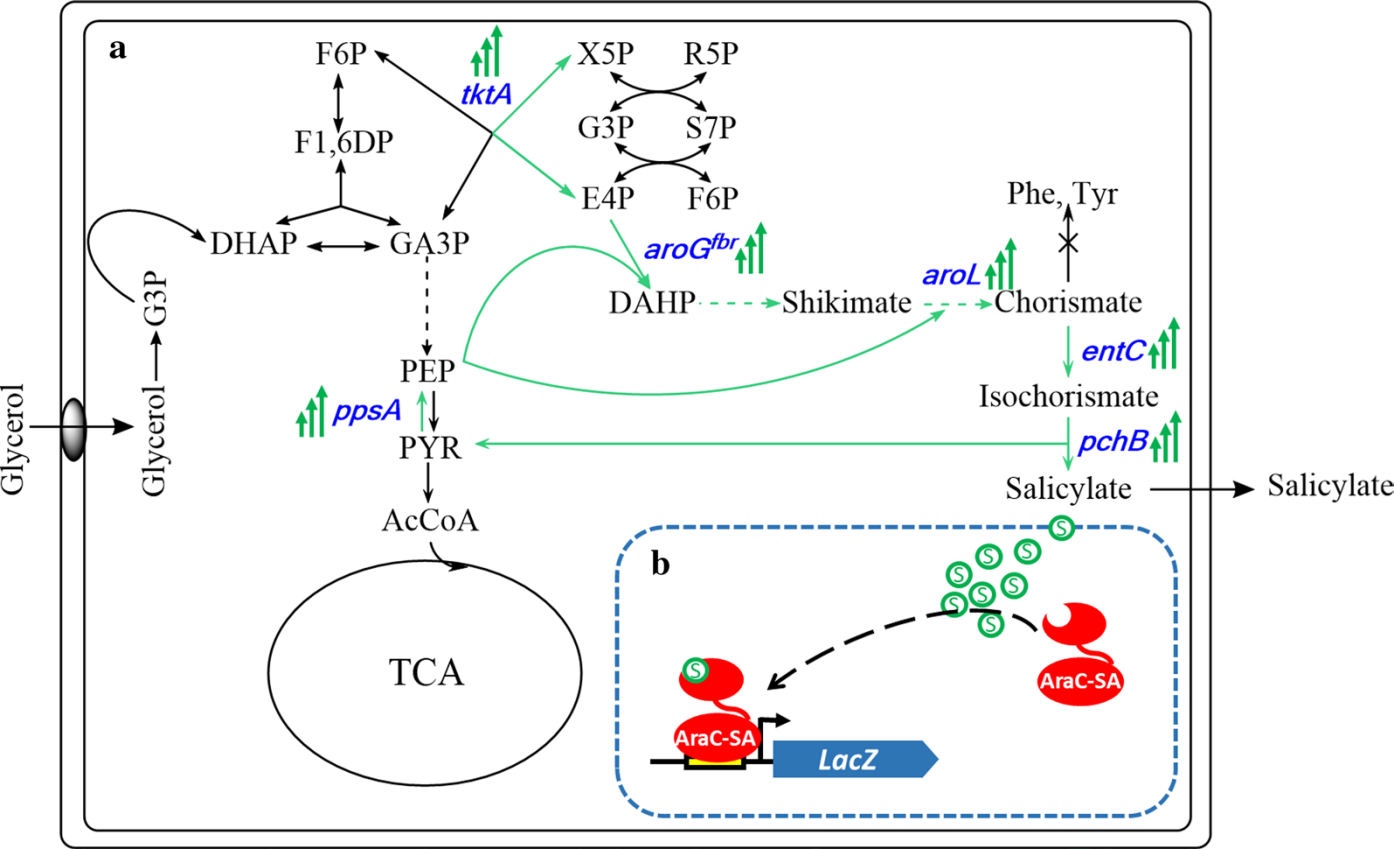
Sensor-based pathway optimization for improving salicylate production. **a** Synthetic pathway for producing salicylate from glycerol. Six genes highlighted in blue encode the key enzymes involved in salicylate biosynthesis: *ppsA* encodes PEP synthetase, which converts pyruvate to PEP. *tktA* encodes a transketolase that increases supply of E4P. DAHP synthase encoded by *aroG*^*fbr*^ catalyzes the aldol condensation to generate DAHP. Shikimate kinase encoded by *aroL* produces chorismate. Isochorismate synthase (encoded by *entC*) and isochorismate pyruvate lyase (encoded by *pchB*) enable the synthesis of salicylate form chorismate. **b** AraC-based salicylate biosensor was used to screen for clones showing enhanced salicylate production

As depicted in Fig. 1, the designed reporter system was applied to optimize the expression level of key enzymes in the shikimate pathway. A series of ribosome binding site (RBS) sequences corresponding to a spectrum of predicted translation rates were designed for each of six genes involved in the heterologous salicylate biosynthesis pathway (Fig. 1a). Screening the combinatorial expression library resulted in isolation of several clones with enhanced salicylate production (up to 123% improvement). Subsequently, screening a random transposon insertion library identified a knock-out gene target that further improved salicylate titer by 27%.

## Results

### Construction of AraC-based sensor system for agar plate-based high-throughput screening

**Fig. 2:**
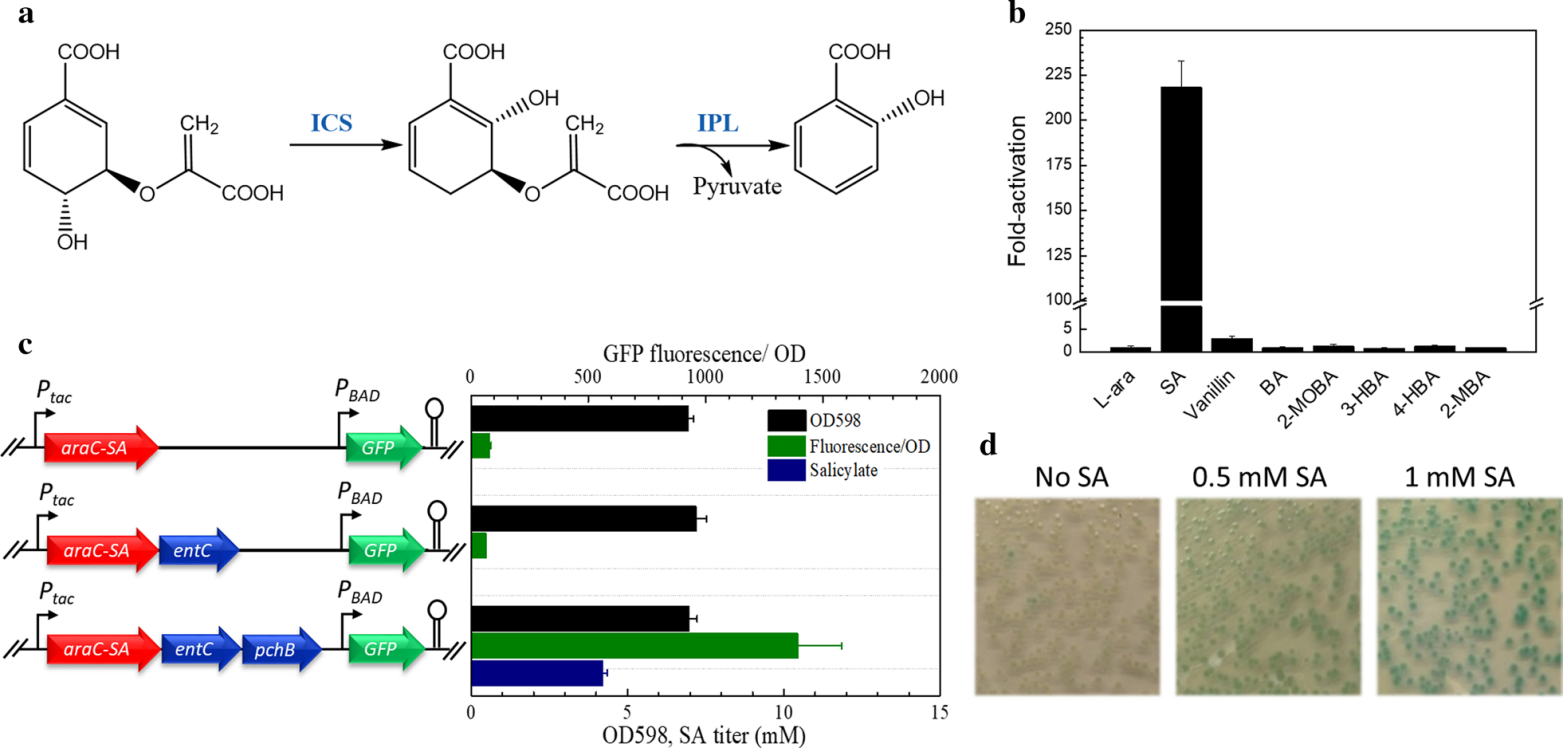
Development of a salicylate-responsive sensor-reporter system. **a** Heterologous pathway for producing salicylate from the native *E. coli* central metabolite chorismate. **b** Fold activation of GFP expression under control of AraC-SA/P_BAD_, in the presence of 5 mM salicylate (SA) and similar compounds (l-ara: l-arabinose (inducer for wild-type AraC); BA: benzoic acid; 2-MOBA: 2-methoxybenzoic acid; 3-HBA: 3-hydroxybenzoic acid; 4-HBA: 4-hydroxybenzoic acid; and 2MBA: 2-methylbenzoic acid. Reported data represents mean ± std. dev., from four biological replicates. **c** Response of AraC sensor in *E. coli* strain QH4 carrying plasmid pGF29-SA (neither *entC* nor *pchB* is expressed), plasmid pFG29-SA-entC (*entC* but not *pchB* are expressed), and plasmid pFG29-SA-EP (enabling salicylate production). **d** Colorimetric colony assay using the designed salicylate sensor system (SQ22 expressing AraC-SA growing on LB-agar plates containing X-GAL and different concentrations of salicylate)

AraC variant “AraC-SA” (P8G, T24L, H80G, Y82I and H93R) responds to salicylate, as described previously [20]. To assess the specificity of this sensor, we compared the response (fold activation of GFP expression under the control of promoter *P*_*BAD*_) to salicylate with that of several other similar molecules (substituted benzoates) (Fig. 2b). A concentration of 5 mM was used to compare compounds, as this amount is relevant to screening salicylate production, without major growth inhibition. Salicylate induces GFP expression over 200-fold, while there is essentially no response to the other compounds tested. The complete AraC-SA salicylate dose response is provided as Additional file 1.

To confirm that AraC-SA does not also respond to compounds upstream in the salicylate biosynthetic pathway (namely chorismate and isochorismate), strains expressing the full or partial salicylate pathway with the AraC-SA/GFP module were constructed (Fig. 2c). Only the strain expressing the full pathway produced salicylate (~ 4.5 mM after 14 h post-induction), resulting in 15-fold increased GFP fluorescence. Strains without the complete pathway exhibited only basal fluorescence signal. The AraC-SA sensor/reporter thus responds specifically to salicylate, at least in the context of salicylate biosynthesis in recombinant *E. coli*.

In using our sensor/reporter system to screen libraries for mutants with enhanced salicylate production, a colony-based screen was developed [21]. This approach provides a simple means of spatially isolating individual mutants and preventing “cheaters” from responding to salicylate produced and secreted by other mutants. To that end, a single copy of *lacZ* under control of *P*_*BAD*_ was integrated into the salicylate production host strain (QH4) [14], resulting in reporter strain SQ22 for library screening. As shown in Fig. 2d, in the presence of X-gal (5-bromo-4-cholo-3-indolyl β-d-galactopyranoside), the colony’s blue color intensity resulting from LacZ-catalyzed X-gal hydrolysis correlates with the salicylate concentration. Further validation of this colony screen for isolating clones with enhanced salicylate production is provided in the (Additional file 2).

### Salicylate biosynthetic pathway RBS library construction and screening

**Fig. 3:**
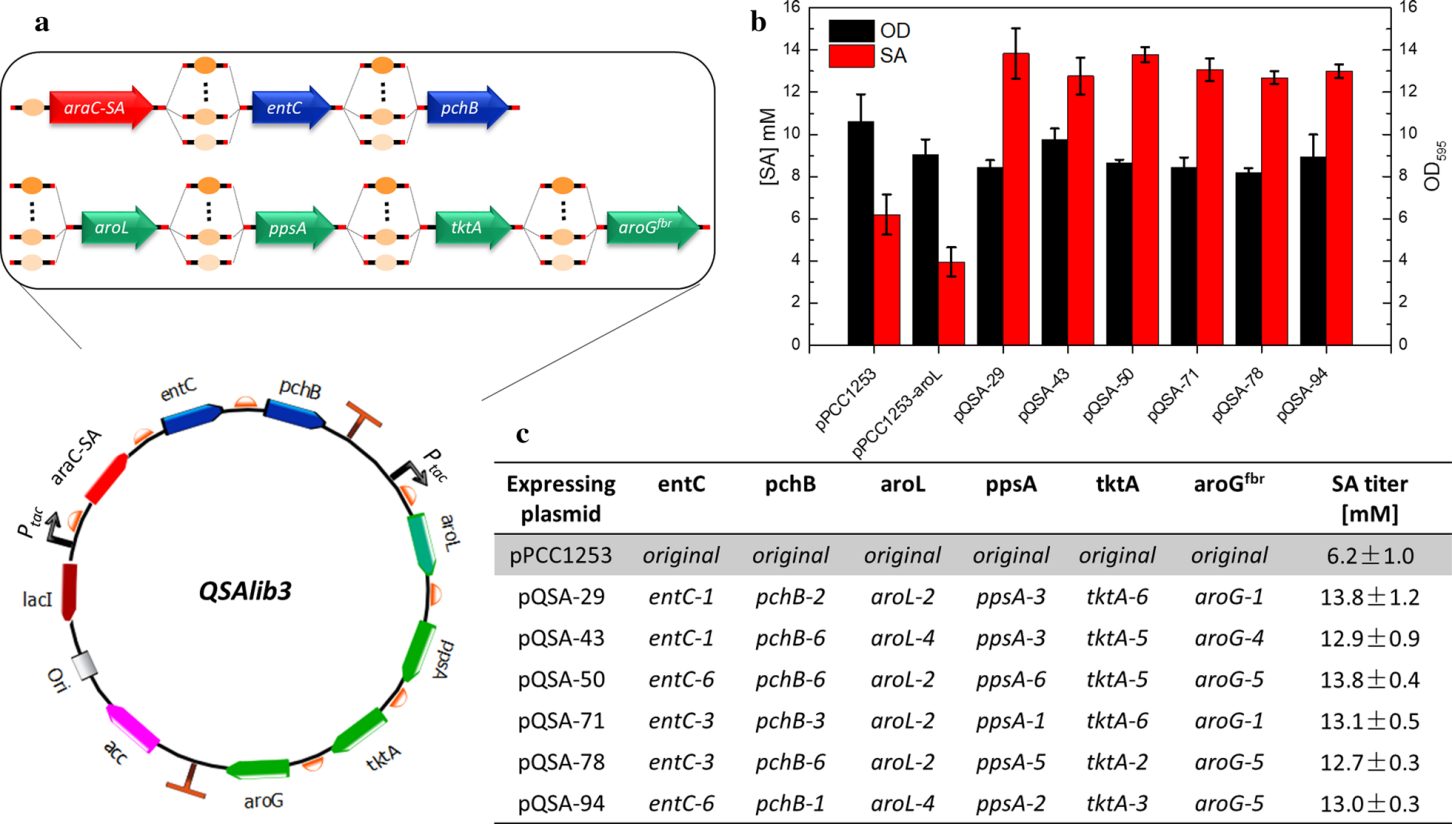
Implementation of sensor-reporter screening to optimize expression of salicylate pathway genes. **a** Schematic of RBS library plasmid constructs. Six RBS sequences ranging from weakest (“gene-1”) to strongest (“gene-6”) calculated translation initiation rates were placed upstream of each pathway gene on plasmid pPCC1253, also containing the AraC-SA sensor. **b** Cell density and salicylate titers measured from the top clones identified from screening. **c** List of RBS combinations for each gene from the top six selected clones. Reported data represents mean ± std. dev., from four independent experiments. The corresponding predicted TIRs are shown in an analogous table in the (Additional file 3). Note that TIRs calculated for a specific gene are relative numbers for predicting the targeted protein relative expression level, and the actual protein expression level is affected by other factors as well. Hence it should not be considered constructive to compare calculated TIRs for different genes

Given the known metabolic burden imposed by gene overexpression, and potential toxicities or enzyme inhibition resulting from accumulation of pathway intermediates [22, 23], we reasoned that one approach to improving salicylate production in recombinant *E. coli* is to fine-tune the relative expression levels of the six key biosynthetic pathway genes (described above). The RBS calculator [7] was used to design a series of RBS sequences resulting in a range of predicted translation initiation strengths for the six targeted genes (*entC*, *pchB*, *aroL*, *ppsA*, *tktA*, *aroG*^*fbr*^) (refer to Additional file 3 for details). These six genes with varied RBS sequences were assembled combinatorially into plasmid pPCC1244, leading to pathway library “QSAlib3” composed of theoretically 38,880 unique members (Fig. 3a). Strain SQ22 containing a chromosomal *P*_*BAD*_-*lacZ* reporter was transformed with the QSAlib3 library and spread onto large, X-Gal-containing plates for screening. Strain SQ22 is derived from strain QH4 [14] having *pheA* and *tyrA* gene deletions (Table 1), which prevents conversion of chorismate to phenylalanine and tyrosine. Yeast extract (5 g/L) was therefore added to our modified minimal medium to support growth.

**Table 1:** Plasmids and strains used in this study

Plasmids	Relevant features	References
pZE-EP	*entC*, *pchB*, and *luc* genes under control of *P*_*lac*_; Amp^R^	[14]
pCS-APTA	*aroL ppsA*, *tktA*, *aroG*^*fbr*^ under control of *P*_*lac*_; Kan^R^	[14]
pPCC1115-5	CRIM plasmid with *P*_*BAD*_-*lacZ*;;Apr^R^	[21, 29]
pPCC1244	*araC*-SA gene under the control of *P*_*tac*_; Apr^R^	In this study
pPCC1250	*entC*, *pchB* genes under control of *P*_*tac*_; Amp^R^	In this study
pPCC1251	*araC*-SA*, entC*, *pchB* genes under control of *P*_*tac*_; Apr^R^	In this study
pPCC1252	*aroL ppsA*, *tktA*, *aroG*^*fbr*^ genes under control of *P*_*tac*_; Apr^R^	In this study
pPCC1253	*araC*-SA*, entC*, *pchB* genes under control of *P*_*tac*_; *aroL ppsA*, *tktA*, *aroG*^*fbr*^ genes under control of *P*_*tac*_; Apr^R^	In this study
pPCC1253-aroL	*araC*-SA*, entC*, *pchB* genes under control of *P*_*tac*_; *ppsA*, *tktA*, *aroG*^*fbr*^ genes under control of *P*_*tac*_; Apr^R^	In this study
pPCC507	From vector pJA1. Transposable element containing a chloramphenicol resistance gene flanked by IS10 inverted repeat sequences; R6 K replication origin; *tnp* gene encoding a mutant Tn10 transposase with 100-fold lower frequency of insertion at hot spots; Amp^R^	[21, 31]
pFG1	*araC* gene under the control of *P*_*tac*_, *tac*; Apr^R^	[18]
pFG29-SA	*araC*-*SA* gene under the control of *P*_*tac*_; *gfpuv* under control of *P*_*BAD*_ promoter, Apr^R^	[20]
*Strains*		
HF19	BW27786 (∆*araFGH*, ∆*araBAD,* ∆*lacZ*), with *araC* deleted	[16]
QH4	ATCC31884 with *pheA* and *tyrA* disrupted	[14]
SQ18	QH4 with *P*_*BAD*_-*lacZ* reporter integrated into chromosome at HK022 site	In this study
SQ22	SQ18 with *LacZ* in the *lac* operon disrupted	In this study
QH4Δrnd	QH4 with *rnd* gene disrupted	In this study
SQ22Δrnd	SQ22 with *rnd* gene disrupted	In this study

Approximately 100,000 colonies were screened by eye. Rescreening of clones representing the 75 darkest blue colonies was performed by quantifying salicylate production in liquid culture (using strain SQ22 harboring the selected mutant plasmids). Among these, the top six mutant plasmids based on salicylate titer were sequenced (Fig. 3c) and salicylate production using these constructs was further characterized with strain QH4 (Fig. 3b). Two clones produced about 13.8 ± 1.2 mM salicylate, which is > 120% improved relative to strain QH4 overexpressing all six target genes, each with the same “original” RBS sequence (AGGAGA) (plasmid pPCC1253). The time courses of salicylate production and cell density for QH4 + pPCC1253 and QH4 + pQSA-50 (provided in Additional file 4) show that salicylate production occurs primarily after growth has already slowed and culture densities are near maximum.

As shown in Fig. 3c, the RBS sequences appearing in the top six selected clones encompass the full range of weak to strong, for all six genes. This gene expression library is represented on polycistronic operons (Fig. 3a), so translational coupling [24] may at least partly explain the general lack of patterns to RBS strengths for the selected clones. While there are no prominent expression patterns for these genes, we note that most clones contain a relatively strong RBS upstream of *tktA*, and relatively weak RBS sequences upstream of *aroL*. SDS-PAGE analysis of cell lysates (using Coomassie blue staining) confirms these observations, in that strong bands are present for transketolase (though less prominent for the weaker RBSs in pQSA-78 and pQSA-94), and the two relatively stronger RBS sequences for *aroL* (pQSA-43 and pQSA-94) show higher *aroL* expression than the others (Additional file 5). We tested whether further lowering *aroL* expression by removing this gene from the plasmid (construct pPCC1253-*aroL*), and relying on only chromosomal expression of this gene, would further improve salicylate production. This however significantly reduced salicylate production (Fig. 3b). These results demonstrate the challenges of rationally tuning pathway gene expression levels, and the value in using combinatorial approaches to identify improvements.

### Screening a transposon insertion library for further improvements in salicylate production

**Fig. 4:**
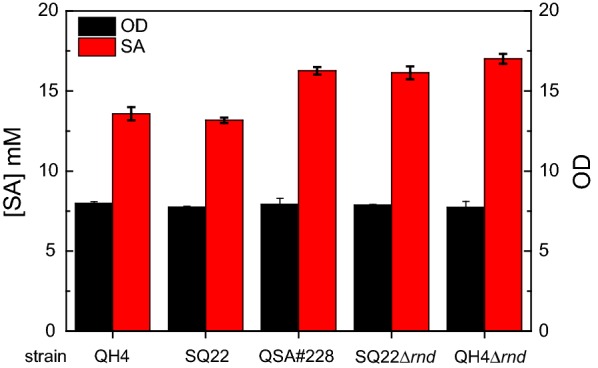
Transposon insertion library screening for improved salicylate production led to the identification of an *rnd* mutation in the host. Red column: Salicylate titer after 48 h fermentation of different host strains expressing plasmid pQSA-50; Black column: cell density of culture after 48 h fermentation. Reported data represents mean ± std. dev., from four independent experiments

We next sought to mutate the host genome and screen for further improvements in salicylate production. A suicide plasmid vector containing a transposable DNA fragment and IPTG-inducible transposase was used to generate the transposon insertion library in strain SQ22 harboring plasmid pQSA-50. The SQ22-transposon insertion library was screened using our AraC-*P*_*BAD*_-*lacZ* sensor-reporter system. After screening ~ 70,000 colonies on plates, 50 selected clones were re-screened in liquid culture for HPLC analysis. Among these, clone QSA#228 consistently produced ~ 16.5 mM salicylate after 48 h induction, which is ~ 27% improved over parent strain SQ22 harboring mutant plasmid pQSA-50 (Fig. 4). Curing the originally selected strain of plasmid, and re-transforming with fresh plasmid pQSA-50 resulted in the same improvement in salicylate titer. Self-formed adaptor PCR (SEFA-PCR) analysis [25] located the transposable element within the *rnd* gene, encoding RNase D. RNase D is an exonuclease involved in the 3′ ribonucleotide processing of tRNA [26]. Direct deletion of *rnd* from the genomes of host strains SQ22 and QH4 resulted in similar improvements in salicylate production. It is not known how changes in protein expression levels due to the *rnd* mutation are beneficial to salicylate biosynthesis.

## Discussion

Salicylate has been widely used as a precursor in the pharmaceuticals and bioplastics industry. Metabolic engineers have successfully synthesized salicylate from recombinant *E. coli* by extending the shikimate pathway [14, 15]. Here we further improved salicylate production in *E. coli* by optimizing expression of key enzymes in the shikimate pathway, with the help of a high-throughput sensor-reporter system. In addition, screening a transposon library with the same reporter system revealed a gene knockout target that further contributes to salicylate biosynthesis. The resulting salicylate titer of 17 mM is the highest reported from shake-flask culture.

It is worth noting that all six of the highest salicylate-producing mutants presented in Fig. 3b (results from RBS library screening) show similar final titers, suggesting a potential “upper limit” to production, at least for this library and under these culture conditions. At least 40 mM glycerol remained in all cultures tested (data not shown), suggesting carbon supply does not limit production. The inhibitory effects of salicylate on growth of strain QH4 are notable even at 5 mM, and more severe as salicylate concentration increases (refer to Additional file 6). However, as noted above, salicylate titers do not exceed 5 mM until culture densities are already near maximum, and maximum culture density is not reduced as a result of increased salicylate production (Fig. 3b and Additional file 4). Nonetheless, salicylate toxicity may contribute to an upper limit on production. Further strain engineering to improve salicylate tolerance and/or export may therefore be promising approaches to further advance the commercial potential of microbial salicylate production.

## Methods

### General

Restriction enzymes, Phusion High-Fidelity DNA polymerase, and T4 DNA ligase were purchased from New England Biolabs (Ipswich, MA). Oligonucleotides were synthesized by Integrated DNA Technologies (Coralville, IA). DNA sequencing was performed at SeqWright (Houston, TX) or Genewiz (South Plainfield, NJ). All chemicals were purchased from Sigma-Aldrich (St. Louis, MO). Molecular biology techniques for DNA manipulation were performed according to standard protocols [27] and all cultures were grown in lysogeny broth (LB). Antibiotics and IPTG were prepared as a 1000× stock solution in purified water and sterile filtered with EMD Millipore Millex-GP syringe driven filters (EMD Millipore, Cat. No. SLGP033RS). The modified NBS medium contains MOPS (50 mM, pH 7.4) and per liter: 10 g glycerol; 2.5 g glucose; 5 g yeast extract; 3.4 g KH2PO_4_; 5.2 g K_2_HPO_4_; 3.3 g (NH_4_)_2_HPO_4_, 0.25 g MgSO_4_·7 H_2_O, 15 mg CaCl_2_·2 H_2_O, 0.5 mg thiamine, and 1 mL of trace metal stock (described by Chin and Cirino [28]). The concentrations of antibiotics used for maintaining the plasmids are as follows: apramycin 50 µg/ml, chloramphenicol 25 µg/ml, ampicillin 100 µg/ml.

### Plasmids

Plasmids and strains used in this study are listed in Table 1. All primers used in this study are listed in Additional file 7. Primers *pPCC1244*-*gib*-*for* and *pPCC1244*-*gib*-*rev* were used to amplify *lacI*-*Ptac*-*araC*-*SA* DNA fragment from pFG29-SA, and the PCR product was assembled with PciI- and XmnI-digested pFG1, resulting in plasmid pPCC1244.

To create plasmid pPCC1250, pZE-EP was double digested with *Bsu36I* and *SphI*, and the digested DNA fragment was subjected to a DNA-blunting reaction. The blunt-ended DNA fragment was self-ligated, resulting in plasmid pPCC1250. A DNA fragment containing *entC* and *pchB* was amplified from pPCC1250 using primers *pPCC1251*-*gib*-*for* and *pPCC1251*-*gib*-*rev2*, and assembled with *XmnI*-digested pPCC1244, resulting in plasmid pPCC1251. A DNA fragment containing *aroL ppsA*, *tktA*, *aroG*^*fbr*^ genes was amplified from pCS-APTA using primers *pPCC1252*-*gib*-*for* and *pPCC1251*-*gib*-*rev2*, and the amplified DNA fragment was assembled with *NdeI*- and *XmnI*-digested pPCC1244, resulting in plasmid pPCC1252. Then, a DNA fragment of *Ptac*-*araC*-*SA*-*entC*-*pchB* was amplified from pPCC1251 using primers *pPCC1253*-*NheI*-*for* and *pPCC1253*-*NotI*-*rev*, and digested with *NheI* and *NotI*. The digested DNA fragment was ligated into pPCC1252 digested with the same restriction enzymes, resulting in plasmid pPCC1253. To remove *aroL* from plasmid pPCC1253, primers *pPCC1251*-*gib*-*for* and *pPCC1253*-*Ptac*-*rev* were used to amplify the *entC*-*pchB* DNA fragment from pPCC1253, and primers *pPCC1253*-*ppsA*-*for* and *pPCC1253*-*tktA*-*rev* were used to amplify the *ppsA*-*tktA* section from pPCC1253. The two amplified PCR products were assembled into *KpnI*/*SphI* double-digested pPCC1253, resulting in plasmid pPCC1253-*aroL.*

### Construction of pathway RBS library

The RBS calculator [7] was used to design six RBS sequences having different translation initiation rates (TIRs) for each gene (see Additional file 1 for the RBS sequences and calculated TIRs). For each gene, the six designed upstream primers containing different RBS sequences were mixed equimolar, resulting in primer mixtures *entC*-*RBSs*-*for*, *pchB*-*RBSs*-*for,* etc., and each primer mixture was used to construct the RBS library. Primers *pFG29_araC_GS_fwd_1* and *AraC*-*gib*-*rev* were used to amplify Ptac-AraC-SA DNA fragment from pPCC1244. Primers *entC*-*RBSs*-*for* and *entC*-*RBS*-*rev* were used to amplify *entC* from pZE-EP, and primers *pchB*-*RBSs*-*for* and pPCC1251-gib-rev2 were used to amplify *pchB* from pZE-EP. Plasmid pPCC1244 was double digested by *BstAPI* and *XmnI*, and the linearized vector was assembled with the three PCR products, resulting in QSALib1, which contains the RBS libraries for *entC* and *pchB* genes.

Primers *pFG29_araC_GS_fwd_1* and *Ptac*-*gib*-*rev* were used to amplify a DNA fragment containing promoter *Ptac*. The *aroL* gene was amplified from pCS-APTA by primers *aroL*-*RBSs*-*for* and *AroL*-*RBS-rev*. Similarly, *ppsA* was amplified from pCS-APTA using primers *ppsA*-*RBSs*-*for* and *ppsA*-*RBS*-*rev*. Overlap extension PCR (OE-PCR) was performed to assemble these three PCR products using primers *pFG29_araC_GS_fwd_1* and *QSAlib2*-*OE123*-*rev*, resulting in DNA fragment QSAlib2-f123. Next, primers *tktA*-*RBSs*-*for* and *tktA*-*RBS*-*rev* were used to amplify *tktA* from plasmid pCS-APTA, and primers *AroG*-*RBSs*-*for* and *pPCC1251*-*gib*-*rev2* were used to amplify *aroG*. *tktA* and *aroG* were assembled by OE-PCR using primers QSAlib2-OE45-for and QSAlib2-OE45-rev, resulting in DNA fragment QSAlib2-f45. pPCC1252 was then double digested with *BstAPI*/*BamHI,* QSAlib2-f123 was double digested with *BstAPI*/*SpeI*, and QSAlib2-f45 was double digested with *SpeI*/*BamHI*. Ligation of these three digest fragments resulted in library QSAlib2.

Finally, primers *pPCC1253*-*NheI*-*for* and *pPCC1253*-*NotI*-*rev* were used to amplify *Ptac*-*araC*-*SA*-*RBS*-*entC*-*RBS*-*pchB* from QSAlib1. The PCR product was digested with *NheI* and *NotI*, and ligated with QSAlib3 digested with the same enzymes, resulting in QSAlib3. QSAlib3 contains the RBS libraries for all six genes. Sanger sequencing of QSAlib3 confirmed proper library construction.

### Strain construction

The strains used in this study are listed in Table 1. Plasmid pPCC1155-5 was integrated into the chromosome of QH4 at site HK022 as described [29]. Removal of FRT-flanked apramycin resistance cassette resulted in strain SQ18. A Phage λ Red disruption method [30] was used to delete *lacZ* from the *lac* operon of strain SQ18, resulting in strain SQ22. Deletion of *rnd* in strains QH4 and SQ22 was similarly performed, resulting in strains QH4∆*rnd* and SQ22*∆rnd*, respectively.

### Sensor-reporter fluorescence assays

Essentially as described [20], 500 μl LB + apramycin in 2-ml-well, 96-well plate was inoculated with strain HF19 harboring pFG29-SA. These starter cultures were incubated for 6 h at 37 °C, 900 rpm, then diluted to OD_595_ = 0.05 in 500 μl “biosensor medium” containing different concentrations of the compound of interest. After 6 h, the cultures were pelleted and washed with an equal volume of phosphate-buffered saline, before measuring OD_595_ and fluorescence (400 nm excitation, 510 nm emission) using plate readers.

### Salicylate production in baffled flasks

A colony of the salicylate-producing strain was used to inoculate 3 ml LB + apramycin, and grown in a test tube for 8 h at 37 °C and 250 rpm. This seed culture was then diluted to OD_595_ = 0.05 into 25 ml modified NBS medium containing apramycin and 250 mM IPTG, in 125 ml baffled flasks. The flasks were shaken at 37 °C and 250 rpm for 48 h, at which time OD_595_ values were measured and salicylate concentrations were analyzed by HPLC.

### Screening the RBS library for improved salicylate producers

Strain SQ22 was transformed with QSAlib3. The outgrowth was transferred into LB + apramycin, and grown at 37 °C, 250 rpm for 12 h. The resulting culture was diluted and spread onto large plates containing modified NBS-agar with IPTG (250 µM), X-Gal (40 µg/ml), and apramycin. After 24 h of incubation, the top 5 blue colonies from each screening plate were picked and streaked onto fresh LB plates supplied with apramycin. The resulting colonies were tested for salicylate production in liquid culture.

### Construction and screening of SQ22 transposon insertion library

Strain SQ22 harboring the highest-producing salicylate plasmid (pQSA-50) was transformed with 1 µg of plasmid pPCC507, and the outgrowth was grown in 1 ml SOB medium supplied with 20 µM IPTG at 37 °C for 1 h. The outgrowth was transferred into 500 ml LB + apramycin and 12.5 µg/ml chloramphenicol, and grown at 37 °C for 12 h. The resulting culture was diluted and plated on modified NBS-agar plates containing IPTG (250 µM), X-Gal (40 µg/ml), apramycin, and 12.5 μg/ml chloramphenicol. Totally 70,000 colonies were screened on 10 screening plates. After 24 h of incubation, the top 5 blue colonies from each screening plate were picked and streaked onto fresh LB plates containing apramycin and 12.5 µg/ml chloramphenicol. The resulting colonies were tested for salicylate production in liquid culture.

### Salicylate quantification by HPLC

500 µl of cell culture was centrifuged at 17,900×*g*, and the supernatant was filtered through a 0.45 µm filter. The salicylate concentration in the filtrate was determined by reverse-phase HPLC using a C18 column on a Shimadzu LC-20AD HPLC system (Kyoto, Japan) equipped with a UV monitor. The elution profile was as follows: Solvent A, 1% (v/v) acetic acid in water; solvent B, 1% (v/v) acetic acid in acetonitrile; gradient: 5–95% B (0–15 min), 95–5% B (15–17 min), 5% B (17–20 min). The column temperature was set to 50 °C. Salicylate eluted around 11.2 min at a flow rate of 1 ml/min. Elution absorbance at 310 nm was monitored and peak areas were converted to sample concentrations based on calibration with pure salicylate.

## Additional files

**Additional file 1.** Salicylate dose response of AraC-SA. Fold-activation of GFP expression under control of P_*BAD*_ and regulated by AraC-SA (strain QH4 + pFG29-SA), in the presence of different concentrations of salicylate. Each data point represents four biological replicates.

**Additional file 2.** Validation of the salicylate sensor-reporter system. Salicylate titers from cultures of randomly picked colonies harboring RBS library plasmid (green) were compared with those from blue colonies picked on screening plates (blue). The average salicylate titer from the eight randomly picked colonies was 7 mM (SD = 3 mM). Meanwhile the average titer from the selected clones was 10 mM (SD = 1 mM). Cells were grown in shake-flask cultures for 48 h, as described in “Methods”.

**Additional file 3.** RBS sequences for each gene and the predicted TIR.

**Additional file 4.** Time-course of salicylate production (culture titer) and cell growth (OD_595_) for select strains. Strains were prepared as described in Methods, and grown in modified NBS medium in baffled, shake-flask cultures, as described.

**Additional file 5.** SDS-PAGE analysis of selected RBS library clones. Lane 2, QH4 blank (no plasmid); lane 3, QH4 with plasmid pPCC1251; lane 4, QH4 with plasmid pPCC1253; lane 5 to lane 10, QH4 with plasmid pQSA-29, pQSA-43, pQSA-50, pQSA-71, pQSA-78, pQSA-94. Predicted molecular weights of the proteins encoded by the genes: *entC*, 42.9 kDa; *pchB*, 14 kDa; *aroL*, 19.2 kDa; *ppsA*, 87.4 kDa; tktA, 72.2 kDa; aroG, 38 kDa; aaC, 29.4 kDa.

**Additional file 6.** Effect of salicylate concentration on growth of host strain QH4. A colony of strain QH4 was used to inoculate 3 ml LB, and grown in a test tube for 8 h at 37 °C and 250 rpm. This seed culture was then diluted to OD_595_ = 0.05 into 500 µl modified NBS medium containing the indicated concentration of salicylate, and grown in 2-ml wells (in a 96-well plate). OD_595_ was recorded every 2 h. The data reported represent the average from two independent experiments.

**Additional file 7.** Primers used in this study.

## Abbreviations

AcCoA: acetyl-CoA
BA: benzoic acid
DHAP: dihydroxyacetone phosphate
DAHP: 3-deoxy-d-arabino-heptulosonate-7-phosphate
E4P: erythrose-4-phosphate
F1,6P: fructose-1,6-phosphate
F6P: fructose-6-phosphate
G6P: glucose-6-phosphate
3-HBA: 3-hydroxybenzoic acid
4-HBA: 4-hydroxybenzoic acid
l-ara: l-arabinose
2-MOBA: 2-methoxybenzoic acid
2MBA: 2-methylbenzoic acid
PEP: phosphoenolpyruvate
PR: pyruvate
RBS: ribosome binding site
R5P: ribose-5-phosphate
SA: salicylate
S7P: sedoheptulose-7-phosphate
TIR: translation initiation rate
X5P: xylulose-5-phosphate
