# Supplementary material for: Biosensor-guided improvements in salicylate production by recombinant Escherichia coli
Source: Microb Cell Fact. 2019 Jan 29;18:18. doi: 10.1186/s12934-019-1069-1 (PMC6350385; doi:10.1186/s12934-019-1069-1)
Supplement: Supplementary file 1 — Additional file 1. Salicylate dose response of AraC-SA. Fold-activation of GFP expression under control of PBAD and regulated by AraC-SA (strain QH4 + pFG29-SA), in the presence of different concentrations of salicylate. Each data point represents four biological replicates. [file 12934_2019_1069_MOESM1_ESM.docx]

**Salicylate dose response of AraC-SA.** Fold-activation of GFP expression under control of P*_BAD_* and regulated by AraC-SA (strain QH4+pFG29-SA), in the presence of different concentrations of salicylate. Each data point represents four biological replicates.
