# Supplementary material for: Biosensor-guided improvements in salicylate production by recombinant Escherichia coli
Source: Microb Cell Fact. 2019 Jan 29;18:18. doi: 10.1186/s12934-019-1069-1 (PMC6350385; doi:10.1186/s12934-019-1069-1)
Supplement: Supplementary file 2 — Additional file 2. Validation of the salicylate sensor-reporter system. Salicylate titers from cultures of randomly picked colonies harboring RBS library plasmid (green) were compared with those from blue colonies picked on screening plates (blue). The average salicylate titer from the eight randomly picked colonies was 7 mM (SD = 3 mM). Meanwhile the average titer from the selected clones was 10 mM (SD = 1 mM). Cells were grown in shake-flask cultures for 48 h, as described in “Methods”. [file 12934_2019_1069_MOESM2_ESM.docx]

**Validation of the salicylate sensor-reporter system.** Salicylate titers from cultures of randomly picked colonies harboring RBS library plasmid (green) were compared with those from blue colonies picked on screening plates (blue). The average salicylate titer from the eight randomly picked colonies was 7 mM (Std. Dev. = 3 mM). Meanwhile the average titer from the selected clones was 10 mM (Std. Dev. = 1 mM). Cells were grown in shake-flask cultures for 48 hours, as described in “Methods”.
