# Supplementary material for: Biosensor-guided improvements in salicylate production by recombinant Escherichia coli
Source: Microb Cell Fact. 2019 Jan 29;18:18. doi: 10.1186/s12934-019-1069-1 (PMC6350385; doi:10.1186/s12934-019-1069-1)
Supplement: Supplementary file 3 — Additional file 3. RBS sequences for each gene and the predicted TIR. [file 12934_2019_1069_MOESM3_ESM.docx]

The designed RBS sequence for each gene and the corresponding predicted TIR (calculated using: RBS calculator https://salislab.net/software/)

| **Gene** | **RBS** | **Target TIR** | **sequence** | **Predicted TIR** |
| --- | --- | --- | --- | --- |
| entC | orginal | NA | gaatgttaaag**aggaga**aaggtacc | 18969.71 |
| entC | entC-1 | 300 | agaatcttaaaggatccaccaagccactcagtagggtaataatg | 284.34 |
| entC | entC-2 | 1000 | agaatcttaaaggatccaccaacggaataagaagactaataatg | 1004.96 |
| entC | entC-3 | 3000 | agaatcttaaaggatccaccaaacgattcacgaggctaataatg | 3081.95 |
| entC | entC-4 | 10000 | agaatcttaaaggatccaccaagttcttaaggagtttaataatg | 10435.19 |
| entC | entC-5 | 30000 | agaatcttaaaggatccaccaaacaaatcaggaggctaataatg | 31858.21 |
| entC | entC-6 | 100000 | agaatcttaaaggatccaccaggtacataaggaggttaataatg | 94670.66 |
| **Gene** | **RBS** | **Target TIR** | **sequence** | **Predicted TIR** |
| pchB | orginal | NA | catatg**aggaga**tatacc | 5527.56 |
| pchB | pchB-1 | 300 | cataaggtaccattcaaccaaccgattaatcagactaataatg | 298.15 |
| pchB | pchB-2 | 1000 | cataaggtaccattcaaccaaaacattcagaagaataataatg | 1000.45 |
| pchB | pchB-3 | 3000 | cataaggtaccattcaaccaatcaagtcaggagtctaataatg | 2999.84 |
| pchB | pchB-4 | 10000 | cataaggtaccattcaaccaaactagtaaggagcataataatg | 10020.97 |
| pchB | pchB-5 | 30000 | cataaggtaccattcaaccaaaaaagttaggaggataataatg | 30456.23 |
| pchB | pchB-6 | 100000 | cataaggtaccattcaaccaaaaaagtaaggaggttaataatg | 75594.4 |
| **Gene** | **RBS** | **Target TIR** | **sequence** | **Predicted TIR** |
| aroL | orginal | NA | ttttcacac**aggaga**tatcat | 4538.68 |
| aroL | aroL-1 | 100 | tttcacaccctaggccatctaggtccttaccagtttaataatg | 100.78 |
| aroL | aroL -2 | 300 | tttcacaccctaggccatctagcgccttaaaaggttaataatg | 285.03 |
| aroL | aroL -3 | 1000 | tttcacaccctaggccatctagggtttcaaaaggataataatg | 956.07 |
| aroL | aroL -4 | 3000 | tttcacaccctaggccatctagggtctcaggagcctaataatg | 2959.61 |
| aroL | aroL -5 | 10000 | tttcacaccctaggccatctagggcttcaggagctaataaatg | 4790.36 |
| **Gene** | **RBS** | **Target TIR** | **sequence** | **Predicted TIR** |
| ppsA | orginal | NA | catatg**aggaga**tatacc | 1099.61 |
| ppsA | ppsA-1 | 100 | catattctagaagacactatataggtttactagattaataatg | 122.96 |
| ppsA | ppsA-2 | 300 | catattctagaagacactatacgaatttagtaggataataatg | 298.15 |
| ppsA | ppsA-3 | 1000 | catattctagaagacactatatagtcttaccaggctaataatg | 1051.22 |
| ppsA | ppsA-4 | 3000 | catattctagaagacactatagttactaagtagaataataatg | 3543.36 |
| ppsA | ppsA-5 | 10000 | catattctagaagacactatacttcgttaagagggtaataatg | 10410.21 |
| ppsA | ppsA-6 | 30000 | catattctagaagacactatacttcttcaggaggctaataatg | 36793.07 |
| **Gene** | **RBS** | **Target TIR** | **sequence** | **Predicted TIR** |
| tktA | orginal | NA | gtcgag**aggaga**tatacc | 3387.43 |
| tktA | tktA-1 | 100 | gtcgagactagtccacaaaaaatctagtccaagctaataaatg | 92.3 |
| tktA | tktA-2 | 300 | gtcgagactagtccacaaaaaattcctcacaagcttaataaatg | 286.57 |
| tktA | tktA-3 | 1000 | gtcgagactagtccacaaaaatcacgtcataagcttaataaatg | 908.18 |
| tktA | tktA-4 | 3000 | gtcgagactagtccacaaaaatactttcaacagcctaataaatg | 2719.5 |
| tktA | tktA-5 | 10000 | gtcgagactagtccacaaaaagtcgctcaggagcataataaatg | 10029.99 |
| tktA | tktA-6 | 30000 | gtcgagactagtccacaaaaaacttctcaggagggtaataaatg | 30898.02 |
| Gene | RBS | Target TIR | sequence | Predicted TIR |
| aroG | orginal | NA | gcatgc**aggaga**tatacc | 4055.56 |
| aroG | aroG-1 | 100 | gcatgcaacgaagaccatcaaaaattcatcagtctaataatg | 84.36 |
| aroG | aroG-2 | 300 | gcatgcaacgaagaccatcaaagctttactagcttaataatg | 285.03 |
| aroG | aroG-3 | 1000 | gcatgcaacgaagaccatcaagtacttagtagtataataatg | 1051.22 |
| aroG | aroG-4 | 3000 | gcatgcaacgaagaccatcaagccctaaataggctaataatg | 2829.37 |
| aroG | aroG-5 | 10000 | gcatgcaacgaagaccatcaatctattaggagcctaataatg | 9975.97 |
| aroG | aroG-6 | 30000 | gcatgcaacgaagaccatcaacagctaaggagggtaataatg | 33545.54 |
