# Supplementary material for: Biosensor-guided improvements in salicylate production by recombinant Escherichia coli
Source: Microb Cell Fact. 2019 Jan 29;18:18. doi: 10.1186/s12934-019-1069-1 (PMC6350385; doi:10.1186/s12934-019-1069-1)
Supplement: Supplementary file 4 — Additional file 4. Time-course of salicylate production (culture titer) and cell growth (OD595) for select strains. Strains were prepared as described in Methods, and grown in modified NBS medium in baffled, shake-flask cultures, as described. [file 12934_2019_1069_MOESM4_ESM.docx]

**Time-course of salicylate production (culture titer) and cell growth (OD_595_) for select strains.** Strains were prepared as described in Methods, and grown in modified NBS medium in baffled, shake-flask cultures, as described.
