# Supplementary material for: Biosensor-guided improvements in salicylate production by recombinant Escherichia coli
Source: Microb Cell Fact. 2019 Jan 29;18:18. doi: 10.1186/s12934-019-1069-1 (PMC6350385; doi:10.1186/s12934-019-1069-1)
Supplement: Supplementary file 5 — Additional file 5. SDS-PAGE analysis of selected RBS library clones. Lane 2, QH4 blank (no plasmid); lane 3, QH4 with plasmid pPCC1251; lane 4, QH4 with plasmid pPCC1253; lane 5 to lane 10, QH4 with plasmid pQSA-29, pQSA-43, pQSA-50, pQSA-71, pQSA-78, pQSA-94. Predicted molecular weights of the proteins encoded by the genes: entC, 42.9 kDa; pchB, 14 kDa; aroL, 19.2 kDa; ppsA, 87.4 kDa; tktA, 72.2 kDa; aroG, 38 kDa; aaC, 29.4 kDa. [file 12934_2019_1069_MOESM5_ESM.docx]

**
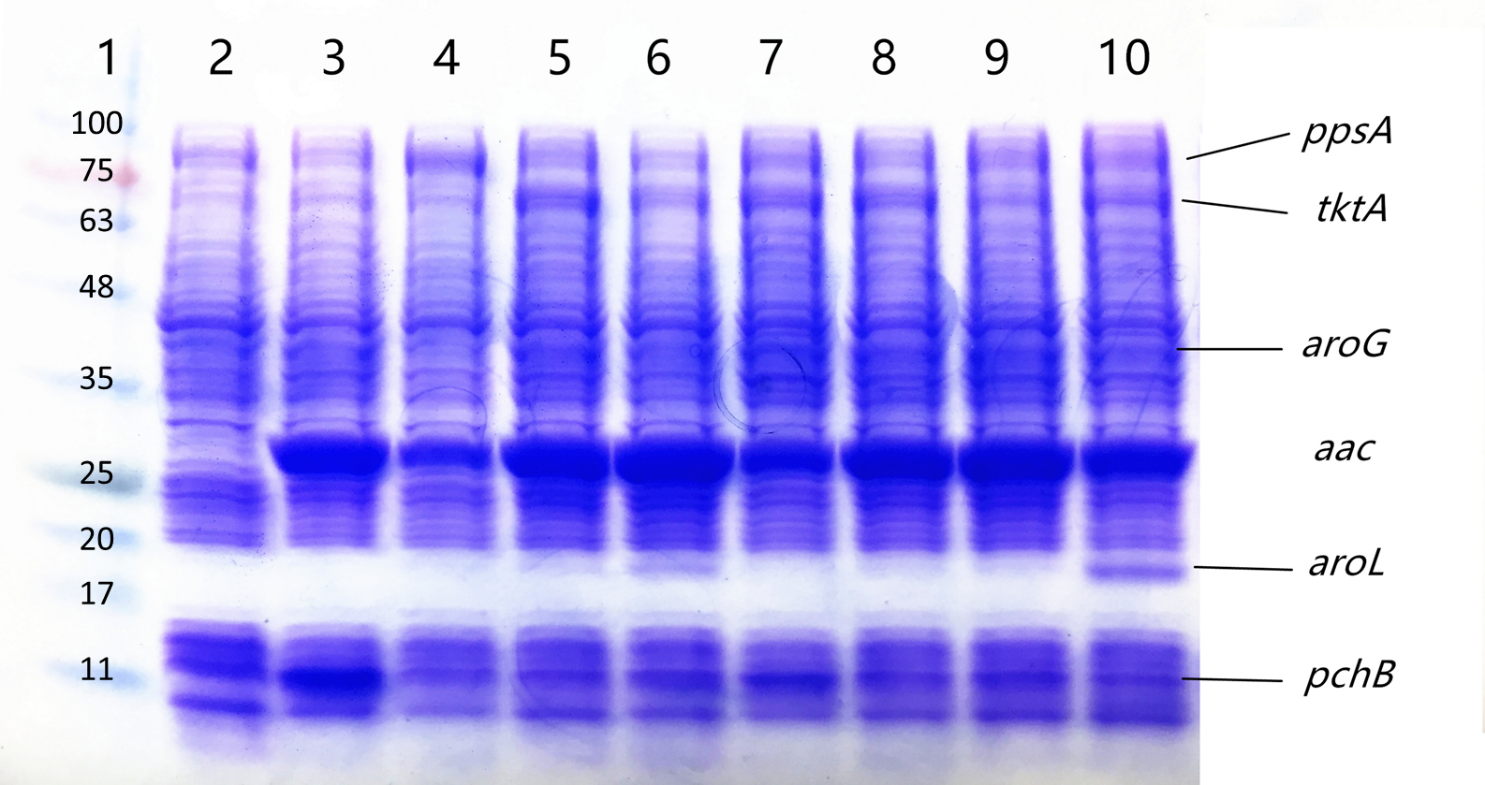
**

**SDS-PAGE analysis of selected RBS library clones.** Lane 2, QH4 blank (no plasmid); lane 3, QH4 with plasmid pPCC1251; lane 4, QH4 with plasmid pPCC1253; lane 5 to lane 10, QH4 with plasmid pQSA-29, pQSA-43, pQSA-50, pQSA-71, pQSA-78, pQSA-94. Predicted molecular weights of the proteins encoded by the genes: *entC*, 42.9kDa; *pchB*, 14kDa; *aroL*, 19.2kDa; *ppsA*, 87.4kDa; *tktA*, 72.2kDa; *aroG*, 38kDa; *aaC*, 29.4kDa.
