# Supplementary material for: Biosensor-guided improvements in salicylate production by recombinant Escherichia coli
Source: Microb Cell Fact. 2019 Jan 29;18:18. doi: 10.1186/s12934-019-1069-1 (PMC6350385; doi:10.1186/s12934-019-1069-1)
Supplement: Supplementary file 6 — Additional file 6. Effect of salicylate concentration on growth of host strain QH4. A colony of strain QH4 was used to inoculate 3 ml LB, and grown in a test tube for 8 h at 37 °C and 250 rpm. This seed culture was then diluted to OD595 = 0.05 into 500 µl modified NBS medium containing the indicated concentration of salicylate, and grown in 2-ml wells (in a 96-well plate). OD595 was recorded every 2 h. The data reported represent the average from two independent experiments. [file 12934_2019_1069_MOESM6_ESM.docx]

**Effect of salicylate concentration on growth of host strain QH4.** A colony of strain QH4 was used to inoculate 3 ml LB, and grown in a test tube for 8 hrs at 37 °C and 250 rpm. This seed culture was then diluted to OD_595_=0.05 into 500 µl modified NBS medium containing different concentrations of salicylate, and grown in 2-ml wells (in a 96-well plate). OD_595_ was recorded every two hours. The data reported represent the average from two independent experiments.
