# Supplementary material for: Biosensor-guided improvements in salicylate production by recombinant Escherichia coli
Source: Microb Cell Fact. 2019 Jan 29;18:18. doi: 10.1186/s12934-019-1069-1 (PMC6350385; doi:10.1186/s12934-019-1069-1)
Supplement: Supplementary file 7 — Additional file 7. Primers used in this study. [file 12934_2019_1069_MOESM7_ESM.docx]

Primers used in this study.

| Primer name | Sequence |
| --- | --- |
| araC-gib-reverse | tggtggatcctttaagattcttatgacaacttgacggctac |
| aroG-RBS1-for | ccaacgaagaccatcaaaaattcatcagtctaataatgaattatcagaacgacgatttac |
| aroG-RBS2-for | ccaacgaagaccatcaaagctttactagcttaataatgaattatcagaacgacgatttac |
| aroG-RBS3-for | ccaacgaagaccatcaagtacttagtagtataataatgaattatcagaacgacgatttac |
| aroG-RBS4-for | ccaacgaagaccatcaagccctaaataggctaataatgaattatcagaacgacgatttac |
| aroG-RBS5-for | ccaacgaagaccatcaatctattaggagcctaataatgaattatcagaacgacgatttac |
| aroG-RBS6-for | ccaacgaagaccatcaacagctaaggagggtaataatgaattatcagaacgacgatttac |
| aroL-RBS1-for | tcacaccctaggccatctaggtccttaccagtttaataatgacacaacctctttttctg |
| aroL-RBS2-for | tcacaccctaggccatctagcgccttaaaaggttaataatgacacaacctctttttctg |
| aroL-RBS3-for | tcacaccctaggccatctagggtttcaaaaggataataatgacacaacctctttttctg |
| aroL-RBS4-for | tcacaccctaggccatctagggtctcaggagcctaataatgacacaacctctttttctg |
| aroL-RBS5-for | tcacaccctaggccatctagggcttcaggagctaataaatgacacaacctctttttctg |
| aroL-RBS-rev | tatagtgtcttctagaatatgtcaacaattgatcgtctg |
| entC-RBS1-for | agaatcttaaaggatccaccaagccactcagtagggtaataatggatacgtcactggct |
| entC-RBS2-for | agaatcttaaaggatccaccaacggaataagaagactaataatggatacgtcactggct |
| entC-RBS3-for | agaatcttaaaggatccaccaaacgattcacgaggctaataatggatacgtcactggct |
| entC-RBS4-for | agaatcttaaaggatccaccaagttcttaaggagtttaataatggatacgtcactggct |
| entC-RBS5-for | agaatcttaaaggatccaccaaacaaatcaggaggctaataatggatacgtcactggct |
| entC-RBS6-for | agaatcttaaaggatccaccaggtacataaggaggttaataatggatacgtcactggct |
| entC-RBS-rev | ttggttgaatggtaccttatgttaatgcaatccaaaaacgtt |
| lacZ-del-for | cataatggatttccttacgcgaaatacgggcagacatggcctgtcaaacatgagaattaa |
| lacZ-del-rev | ggaattgtgagcggataacaatttcacacaggaaacagctgtgtaggctggagctgcttc |
| pchB-RBS1-for | cataaggtaccattcaaccaaccgattaatcagactaataatgctggccttcgacc |
| pchB-RBS2-for | cataaggtaccattcaaccaaaacattcagaagaataataatgctggccttcgacc |
| pchB-RBS3-for | cataaggtaccattcaaccaatcaagtcaggagtctaataatgctggccttcgacc |
| pchB-RBS4-for | cataaggtaccattcaaccaaactagtaaggagcataataatgctggccttcgacc |
| pchB-RBS5-for | cataaggtaccattcaaccaaaaaagttaggaggataataatgctggccttcgacc |
| pchB-RBS6-for | cataaggtaccattcaaccaaaaaagtaaggaggttaataatgctggccttcgacc |
| pFG29_araC_GS_fwd_1 | ataagagacaccggcatact |
| pPCC1244-gib-for | gctggccttttgctcaacatgtcactgcccgctttccag |
| pPCC1244-gib-rev | gagctgcacatgaacccattcttatgacaacttgacggctacatc |
| pPCC1251-gib-for | caagttgtcataagaatgttaaagaggagaaaggtaccatg |
| pPCC1251-gib-rev | gagctgcacatgaaccacaacagataaaacgaaagg |
| pPCC1251-gib-rev2 | gagctgcacatgaacctttgtcctactcaggagagc |
| pPCC1252-gib-for | tttcacacaggagatatcatatgacacaacctctttttctg |
| pPCC1253-NheI-for | aattcgctagccgagctgttgacaattaatc |
| pPCC1253-NotI-rev | aacagctcgcggccgctttgtcctactcaggagagc |
| pPCC1253-ppsA-for | ttcacacaggagatatcatatgtccaacaatggctcg |
| pPCC1253-Ptac-rev | catatgatatctcctgtgtg |
| pPCC1253-tktA-rev | aattcatggtatatctcctgcatg |
| ppsA-RBS1-for | tgacatattctagaagacactatataggtttactagattaataatgtccaacaatggctc |
| ppsA-RBS2-for | tgacatattctagaagacactatacgaatttagtaggataataatgtccaacaatggctc |
| ppsA-RBS3-for | tgacatattctagaagacactatatagtcttaccaggctaataatgtccaacaatggctc |
| ppsA-RBS4-for | tgacatattctagaagacactatagttactaagtagaataataatgtccaacaatggctc |
| ppsA-RBS5-for | tgacatattctagaagacactatacttcgttaagagggtaataatgtccaacaatggctc |
| ppsA-RBS6-for | tgacatattctagaagacactatacttcttcaggaggctaataatgtccaacaatggctc |
| ppsA-RBS-rev | tttttgtggactagtctcgacttatttcttcagttcagc |
| Ptac-gib-rev | ctagatggcctagggtgtgaaatgtgaaattgttatccgctcac |
| QSAlib2-OE123-rev | tttttgtggactagtctcgac |
| QSAlib2-OE45-for | gaaataagtcgagactagtccac |
| QSAlib2-OE45-rev | gagctgcacatgaacctttgtc |
| rnd-del-for2 | gagcgcgcccagtccggcttcggaagattttactgcggatattcctgcaagctgtcaaacatgagaatta |
| rnd-del-rev2 | ccggttaatccggcgttttttttgacgcccactaaagagaaaacaatttggtgtaggctggagctgcttc |
| tktA-RBS1-for | gtcgagactagtccacaaaaaatctagtccaagcttaataatgtcctcacgtaaagagc |
| tktA-RBS2-for | gtcgagactagtccacaaaaaattcctcacaagcttaataaatgtcctcacgtaaagagc |
| tktA-RBS3-for | gtcgagactagtccacaaaaatcacgtcataagcttaataaatgtcctcacgtaaagagc |
| tktA-RBS4-for | gtcgagactagtccacaaaaatactttcaacagcctaataaatgtcctcacgtaaagagc |
| tktA-RBS5-for | gtcgagactagtccacaaaaagtcgctcaggagcataataaatgtcctcacgtaaagagc |
| tktA-RBS6-for | gtcgagactagtccacaaaaaacttctcaggagggtaataaatgtcctcacgtaaagagc |
| tktA-RBS-rev | ttgatggtcttcgttggatgcttacagcagttcttttg |
